# Contribution of *FGFR1* Variants to Craniofacial Variations in East Asians

**DOI:** 10.1371/journal.pone.0170645

**Published:** 2017-01-27

**Authors:** Mohamed Adel, Tetsutaro Yamaguchi, Daisuke Tomita, Takatoshi Nakawaki, Yong-Il Kim, Yu Hikita, Shugo Haga, Masahiro Takahashi, Mohamed A. Nadim, Akira Kawaguchi, Mutsumi Isa, Walid H. El-Kenany, Abbadi A. El-Kadi, Soo-Byung Park, Hajime Ishida, Koutaro Maki, Ryosuke Kimura

**Affiliations:** 1 Department of Orthodontics, Showa University, Tokyo, Japan; 2 Department of Orthodontics, Suez Canal University, Ismailia, Egypt; 3 Department of Orthodontics, Dental Research Institute, Pusan National University Dental Hospital, Yangsan, South Korea; 4 Department of Human Biology and Anatomy, Graduate School of Medicine, University of the Ryukyus, Okinawa, Japan; 5 Department of Orthodontics, Alexandria University, Alexandria, Egypt; Boston University Henry M Goldman School of Dental Medicine, UNITED STATES

## Abstract

*FGFR1* plays an important role in the development of the nervous system as well as the regulation of the skeletal development and bone homeostasis. Mutations in *FGFR1* genes affect skull development, specifically suture and synchondrosis, resulting in craniosynostosis and facial abnormalities. We examined subjects with normal skull morphology for genetic polymorphisms that might be associated with normal craniofacial variations. Genomic DNA was obtained from 216 Japanese and 227 Korean subjects. Four *FGFR1* SNPs, namely, rs881301, rs6996321, rs4647905, and rs13317, were genotyped. These SNPs were tested for association with craniofacial measurements obtained from lateral and posteroanterior cephalometries, in which principle component analysis was performed to compress the data of the craniofacial measurements. We observed that SNPs rs13317 and rs6996321 were correlated with the overall head size and midfacial development, indicating that *FGFR1* SNPs played crucial roles in the normal variation of human craniofacial morphology. Subjects with the derived alleles of SNPs rs13317 and rs6996321 had a small face and a facial pattern associated with a retruded midface and relatively wide-set eyes. These facial features were similar to but were milder than those of individuals with Pfeiffer syndrome, which is caused by a dysfunctional mutation in *FGFR1*.

## Introduction

The human face not only hosts the most important sensory organs but also is the main symbol of expression, appearance, communication, and identification. Facial shape is one of the most important characteristics in developing a self-image and self-esteem [1,2]. Assessment of the contribution of the genome to the morphogenesis and patterning of facial traits inherited from parents is an intriguing topic in social, biological, and medical sciences. Human craniofacial morphology is highly heritable; however, environmental factors also play an important role in craniofacial development [3,4].

The fibroblast growth factor receptor 1 gene (*FGFR1*), which is located at 8p11.1, contains 19 exons spanning 55-kb DNA and encodes at least 9 isoforms of FGFR1 [5]. FGFR1 plays an important role in the development of the nervous system and regulation of skeletal development and bone homeostasis [6,7]. Many developmental disorders affecting the craniofacial area result from dysfunctional mutations in genes encoding FGFs and FGFRs. Identification of these mutations provides insights into the role of FGF/FGFR signaling in normal craniofacial development [8,9]. Mutations in genes involved in FGR/FGFR signaling are associated with craniosynostosis and related syndromes such as Jackson—Weiss, Muenke, Crouzon, Beare—Stevenson, Apert, and Pfeiffer syndromes, thus highlighting the role of these genes in skull development, especially suture and synchondrosis [10–12]. The role of FGFR1 in intramembranous bone development is important for understanding its association with craniofacial phenotypic variability. *FGFR1* is highly expressed in midfacial membranous ossification sites. Pfeiffer syndrome is caused by a mutation in *FGFR1* Ig-like domain II-III linker region (*FGFR1*^P252R^). Patients with Pfeiffer syndrome show many characteristic features that affect the midface and the bones of the skull. These features result from coronal synostosis with or without sagittal synostosis. Abnormal growth of the skull bone in these patients leads to bulging and wide-set eyes, high and prominent forehead, underdeveloped upper jaw, and beaked nose [13–15].

Normal variants of *FGFR1* exert lesser effects on its expression and function than mutations in *FGFR1*, which result in a wide range of phenotypes. Coussens and van Daal (2005) studied the association between *FGFR1* variants and craniofacial morphology in non-diseased populations and identified 17 SNPs that were associated with cephalic index and specific facial phenotypes. Their results showed a significant association between rs4647905 and decreased cephalic index in all the populations examined, with a high correlation observed in Asian and female populations [5]. Gòmez-Valdès et al. (2013) examined the association between *FGFR1* SNPs rs4647905, rs2304000, rs2293971, rs3213849, and rs930828 and various cephalometric measurements in Amerindians and related populations. They concluded that *FGFR1* SNPs, particularly rs4647905, played an important role in normal human skull variation by affecting head length and hence the cephalic index [16]. Hünemeier et al. (2014) determined whether *FGFR1* variants affected the pattern and level of head morphological integration (MI) in a Native American population and an admixed population from Mexico and determined the underlying mechanisms. Their results showed that individuals with a derived allele of rs4647905 had significantly greater levels of head MI, indicating that *FGFR1* affected the development of the human head [17]. Moreover, Nonsyndromic cleft lip and palate (NSCL/P) are common defects in the craniofacial area. Cephalometric studies showed unusual facial features particularly in the mid-facial region for those individuals affected by NSCL/P or even non-affected relatives [18,19]. Therefore, another clue regarding the crucial role of *FGFR1* in craniofacial morphogenesis has been obtained from studies suggesting an association between *FGFR1* variants and nonsyndromic clefting [20–22].

Allele frequency and linkage disequilibrium levels are different among the different populations, which happens for several reasons related to the evolutionary history of each population [16]. Therefore, it will be of major interest to evaluate the findings of the previous studies concerned about the contribution of the *FGFR1* polymorphisms to normal craniofacial variation in other human populations. In the present study, we examined the contribution of *FGFR1* SNPs to variations in normal craniofacial morphology by obtaining cephalometric images of 216 Japanese and 227 Korean subjects.

## Materials and Methods

Craniofacial data and genomic DNA were obtained from 216 Japanese and 227 Korean subjects. The Japanese subjects included 43 men and 173 women with age ranging from 18–57 (mean age = 25.6, standard deviation = 6.5), and the Korean subjects included 132 men and 95 women with age ranging from 18–49 (mean age = 26.2, standard deviation = 4.8). The Japanese subjects were patients from Tokyo who underwent orthodontic treatment at an orthodontic clinic in Showa Dental Hospital, whereas the Koreans subjects were healthy volunteers recruited from Pusan, South Korea. Subjects with congenital disorders such as cleft lip and palate or with general physical diseases were excluded from the study. DNA was extracted from the saliva of all the subjects. All the subjects provided written informed consent, and the study was approved by the Ethics Committee and other related committees of the Showa University Dental Hospital (IBR number 108), Pusan National University (IBR number PNUH-2010-1-1) and the University of the Ryukus (IBR number 120).

### Genotyping and sequencing

Oragene DNA kit (DNA Genotek; Kanata, Ontario, Canada) was used for saliva collection, storage, and DNA purification, according to the manufacturer's recommendations. Briefly, the subjects were refrained from eating and drinking for 30 minutes and were asked to spit into a collection vial until an indicated mark (3 mL). The collected saliva was stored at room temperature before DNA extraction. Four *FGFR1* SNPs, namely, rs881301, rs6996321, rs4647905, and rs13317, were genotyped by performing DigiTag2 assay (Nishida et al. 2007) [23]. Allele frequencies were determined for both the populations (Table 1). Haplotypes were determined using PHASE2.0 (Stephens and Donnelly, 2003) [24]. Linkage disequilibrium (LD) coefficients (Dʹ or r-squared) were calculated using Haploview (Barrett et al. 2005) [25] (Table 1).

**Table 1:** Allele frequencies and LD coefficients of *FGFR1* SNPs.

#rs	Chr: position	Location	Alleles		Derived allele frequency		LD coefficients (Dʹ or r-squared; upper right, Japanese; lower left, Korean)			
			Ancestral	Derived	Japanese	Korean	rs881301	rs6996321	rs4647905	rs13317
rs881301	Chr8: 38474800	5ʹ flanking	T	C	0.320	0.353	-	0.68/0.17	0.41/0.03	0.46/0.06
rs6996321	Chr8: 38464828	Intronic	G	A	0.434	0.433	0.59/0.14	-	0.72/0.30	0.49/0.18
rs4647905	Chr8: 38415024	Intronic	G	C	0.321	0.369	0.18/0.01	0.62/0.30	-	0.96/0.71
rs13317	Chr8: 38411996	3ʹ UTR	C	T	0.624	0.576	0.23/0.026	0.45/0.19	1.00/0.82	-

### Craniofacial measurements

**Fig 1:**
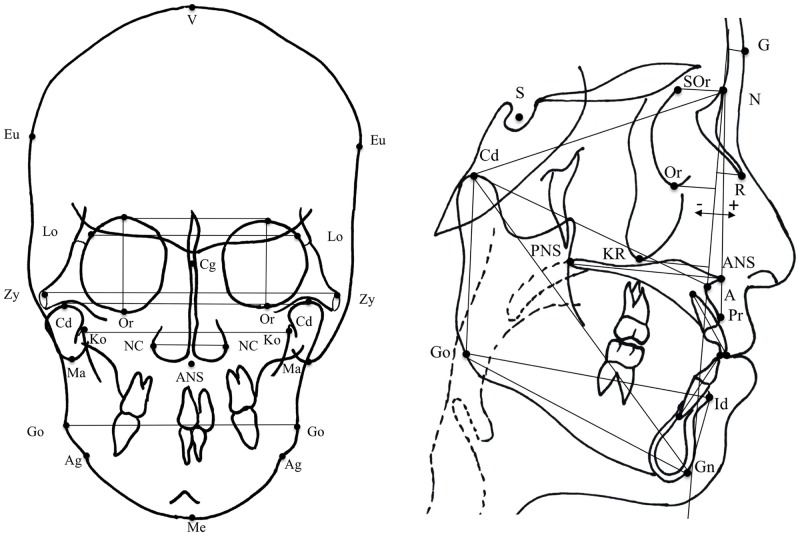
Lateral and posteroanterior cephalometric tracing showing the landmarks used to obtain craniofacial measurements. (V) Vertex, (Eu) eunion, (Lo) latero-orbitale, (Or) orbitale, (Zy) zygion, (Cd) condylion, (Ko) Koronoid, (Ma) mastoid, (NC) nasal cavity, (Cr) crista galli, (ANS) anterior nasal spine, (Go) gonion, (Ag) antegonion, (Me) menton, (G) glabella, (N) nasion, (S) sella turcica, (SOr) supra orbitale, (R) rhinion, (KR) key ridge, (Pr) prosthion, (A) point A, (PNS) posterior nasal spine, (Id) infradentale, (Gn) gnathion. The NA plane was used as a reference to measure the anteroposterior position of G, SOr, R, Or, and KR, with positive and negative values indicating whether the landmark is in an anterior and posterior direction, respectively, from the NA plane.

Lateral and posteroanterior cephalograms of the skull for the Japanese and the Korean subjects were obtained from Showa Dental Hospital and School of Dentistry of Pusan National University, respectively. Cephalograms were taken with the head in a natural position. The head was fixed by fitting the ear rods of the cephalostat in the external auditory meatus. Teeth were held in centric occlusion and the lips were in resting position. Trained technicians took all the radiographs. The radiographic magnification ratio was 1.1. The radiographs of the Japanese subjects were taken using the same machine in Showa Dental Hospital and have the same dot per inch (DPI) value. The DPI value of the lateral and posteroanterior cephalograms was 300. The lateral and the posteroanterior cephalograms of the Korean subjects were obtained using different machines from different radiographic centers in Pusan area and then sent to the School of Dentistry of Pusan National University, thus having different DPI values. The DPI value of the cephalograms of the Korean subjects was 800 (21 subjects), 720 (115 subjects), 600 (39 subjects), 400 (41 subjects), 253 (11 subjects) and 224 (185 subjects). Craniofacial morphology was analyzed by one investigator in Showa Dental Hospital by plotting facial landmarks on posteroanterior and lateral cephalograms by using ImageJ software (version 1.48; Wayne Rasband, National Institute of Health, USA) to obtain their coordinates. Distances between the landmarks were also calculated using ImageJ. The measurements obtained from ImageJ software were changed from pixels to millimeters according to the DPI of each cephalograms using the formula [millimeters = pixels*25.4/DPI]. Fifteen measurements of the cranium and nine measurements of the mandible were obtained from the cephalograms (S1 Table, Fig 1). To investigate intra-operator error, 25 lateral and posteroanterior cephalograms were chosen randomly and re-traced in separate sessions with a two-week interval under identical conditions. Measurement error was estimated according to Dahlberg’s formula (S2 = ∑d2/2n) [26,27]. Positive and negative values of distances measured from Nasion-Point A (NA) plane to other points indicated whether the point was ahead or behind the NA line, respectively. For example, orbitale-NA plane and key ridge-NA plane always had negative values, with the absolute value indicating the distance. Orbital height was calculated by averaging left and right values.

### Statistical analysis

**Fig 2:**
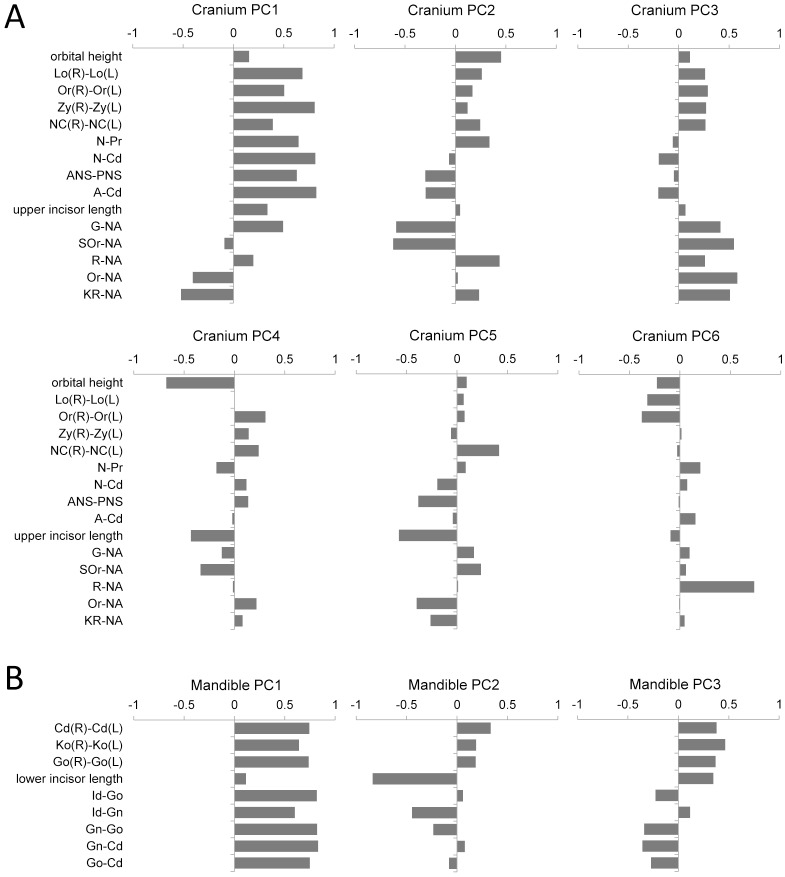
PC loading for each PC. (A) PCA of cranial measurements. (B) PCA of mandibular measurements.

Principal component analysis (PCA) was performed to improve the exploration and visualization of morphological variations by decreasing the dimensionality of obtained data. Metric data of craniofacial morphology was standardized for both the populations, and these standardized data were used to create a correlation coefficient matrix. PCA was performed by using the correlation coefficient matrix, and eigenvalues, eigenvectors and PC scores were obtained. PCA was applied separately to the cranial measurements and to the mandibular measurements (Fig 2). Lateral and the posteroanterior cephalograms were used to load each cranial and mandibular PCA with various measurements in order to detect any variation in craniofacial morphology in all directions. The PCA of the cranial measurements contained measurements indicating the orbital height and protrusion, distance between the orbits, facial width, nasal width and protrusion, maxillary dentoalveolar proturusion, maxillary length and prognathism and forehead protrusion, whereas the PCA of the mandibular measurements implicated measurements indicating the inter-condylar and inter-coroniodal width, mandibular width, lower incisor length, mandibular dentoalveolar protrusion, mandibular body length and mandibular ramus height. Moreover, correlation between cranial and mandibular PCs was examined. Multiple regression analyses were performed to examine the association of *FGFR1* SNPs with PC scores, with sex and population being used as covariates when they were associated with the focal PC. Sex and population were used as covariates in order to control the unexplained variation that may occur from different populations in the sample, which may have different norms of the craniofacial measurements as well as the use of male and female subjects. Single and stepwise multiple SNP models in which genotypes were indicated using the number of derived alleles were examined for each PC. In the stepwise multiple SNP model, the four SNPs were used as explanatory variables at the beginning and were narrowed down later. When two or more SNPs remained after the stepwise procedure, additive multiple SNP and haplotype models were used. In the additive multiple SNP model, the number of effective alleles was added for multiple SNPs. In the haplotype model, haplotype counts in the individual were used as explanatory variables, with the ancestral haplotype as a reference. Furthermore, the 4 SNPs were tested for their association with the ratio |Or-NA|/Or-Or, which represent the relation between the facial retrusion and facial width.

## Results

Craniofacial measurements of the Japanese and Korean subjects included in the study are summarized in S1 Table. We found that Korean subjects had a larger size, on average, than the Japanese subjects in both the cranial and mandibular measurements. PCA of cranial measurements provided an eigenvalue of 4.54 for the first PC (PC1), which contributed to 30.3% of total variance. The cranial PC1 was positively associated with most of the linear measurements of the cranium, which represent the facial width, height and protrusion. PCA of mandibular measurements provided an eigenvalue of 4.47 for PC1, which occupied 49.72%. The mandibular PC1 was positively associated with most of the linear measurements of the mandible, which represent the mandibular width, mandibular ramus height and mandibular body length. Therefore, the cranial PC1 and mandibular PC1 could be interpreted as a size related components. The top six PCs for the cranium and the top three PCs for the mandible had eigenvalues more than or approximately equal to 1, explaining >70% variance in total (S2 and S3 Tables). These PCs were used in subsequent analyses. Significant correlations were observed in some combinations between cranial and mandibular morphological components (S4 Table).

Allele frequencies of the four *FGFR1* SNPs in the Japanese and Korean subjects are presented in Table 1. LD coefficients, i.e., Dʹ and r-squared values, indicated that SNPs rs4647905 and rs13317 were in strong LD with each other (Table 1). The association between the SNPs and cranial and mandibular PC scores was examined using several regression analysis models. Regression analyses by using cranial PC scores indicated a significant correlation of SNPs rs13317 and rs6996321 with PC1 and PC3. The stepwise multiple SNP model showed that SNPs rs13317 and rs6996321 were independently associated with PC1 and PC3. However, the single SNP model showed that only rs13317 was significantly associated with PC1. Regression analyses by using mandibular PC scores indicated that rs4647905 was negatively associated with PC2. Moreover, a significant association was found between the SNP rs13317 and the ratio of the distance between the eyes and the midface retrusion (|Or-NA|/Or-Or) (Table 2).

**Table 2:** Regression analysis to determine the association of FGFR1 SNPs with craniofacial morphological variation in Japanese and Korean subjects.

Outcome variable	Model	Explanatory variable(s) [values]	β1	P1	β2	P2	β3	P3	Covariate(s)
Cranium PC1	Single SNP	X1 = rs881301 [0,1,2]	-0.015	6.5.E-01					Sex, Population
	Single SNP	X1 = rs6996321 [0,1,2]	-0.040	2.2.E-01					Sex, Population
	Single SNP	X1 = rs4647905 [0,1,2]	0.047	1.5.E-01					Sex, Population
	Single SNP	X1 = rs13317 [0,1,2]	**-0.067**	**3.9.E-02**					Sex, Population
	Stepwise multiple SNPs	X1 = rs6996321 [0,1,2], X2 = rs13317 [0,1,2]	**-0.072**	**4.4.E-02**	**-0.096**	**8.1.E-03**			Sex, Population
	Additive multiple SNPs	X1 = rs6996321+rs13317 [0,1,2,3,4]	**-0.083**	**1.1.E-02**					Sex, Population
	Haplotypes*	X1 = H01 [0,1,2], X2 = H10 [0,1,2], X3 = H11 [0,1,2]	-0.096	6.6.E-02	-0.068	1.7.E-01	**-0.110**	**9.7.E-03**	Sex, Population
Cranium PC3	Single SNP	X1 = rs881301 [0,1,2]	0.013	7.8.E-01					Sex
	Single SNP	X1 = rs6996321 [0,1,2]	0.069	1.6.E-01					Sex
	Single SNP	X1 = rs4647905 [0,1,2]	-0.057	2.5.E-01					Sex
	Single SNP	X1 = rs13317 [0,1,2]	0.093	5.6.E-02					Sex
	Stepwise multiple SNPs	X1 = rs6996321 [0,1,2], X2 = rs13317 [0,1,2]	**0.137**	**1.1.E-02**	**0.168**	**2.1.E-03**			Sex
	Additive multiple SNPs	X1 = rs6996321+rs13317 [0,1,2,3,4]	**0.163**	**8.7.E-04**					Sex
	Haplotypes*	X1 = H01 [0,1,2], X2 = H10 [0,1,2], X3 = H11 [0,1,2]	0.118	1.3.E-01	0.087	2.4.E-01	**0.190**	**2.8.E-03**	Sex
Mandible PC2	Single SNP	X1 = rs881301 [0,1,2]	-0.033	4.4.E-01					Sex, Population
	Single SNP	X1 = rs6996321 [0,1,2]	-0.007	8.7.E-01					Sex, Population
	Single SNP	X1 = rs4647905 [0,1,2]	**-0.101**	**2.3.E-02**					Sex, Population
	Single SNP	X1 = rs13317 [0,1,2]	0.078	7.3.E-02					Sex, Population
|Or-NA|/Or-Or	Single SNP	X1 = rs6996321 [0,1,2]	-0.045	3.5.E-01					Sex, Population
	Single SNP	X1 = rs13317 [0,1,2]	**-0.119**	**1.4.E-02**					Sex, Population
	Stepwise multiple SNPs	X1 = rs6996321 [0,1,2], X2 = rs13317 [0,1,2]	-0.104	5.0.E-02	**-0.172**	**1.3.E-03**			Sex, Population
	Additive multiple SNPs	X1 = rs6996321+rs13317 [0,1,2,3,4]	**-0.147**	**2.6.E-03**					Sex, Population
	Haplotypes*	X1 = H01 [0,1,2], X2 = H10 [0,1,2], X3 = H11 [0,1,2]	**-0.193**	**1.3.E-02**	-0.143	5.2.E-02	**-0.180**	**4.4.E-03**	Sex, Population

*Haplotypes comprising two SNPs (rs6996321 and rs13317) were tested, with H00 as the reference haplotype.

Hij indicates a haplotype bearing an i allele at rs6996321 and a j allele at rs13317 (i and j are equal to 0 if the allele is ancestral; otherwise).

Cranial PC1 represented a variation in the overall size of the face, and cranial PC3 represented a variation in midfacial protrusion. High PC3 score was associated with less protrusion of the midface and large facial width in the orbit and cheek region (Fig 2). The number of derived alleles of SNPs rs13317 and rs6996321 was negatively associated with cranial PC1 and was positively associated with PC3 (Table 2), indicating that derived alleles were associated with a small face and midfacial retrusion. Mandibular PC2 was negatively correlated with lower incisor length (Fig 2). Therefore, we examined the association of rs4647905 with lower incisor length and observed a significant association (β = 0.121, P = 0.014), indicating that the derived allele of rs4647905 was associated with long lower incisors.

## Discussion

Because FGF ligands and their receptors play important roles in craniofacial development, especially suture and synchondrosis, variations in genes encoding these ligands and their receptors can affect their expression and functions, thus exerting various effects on craniofacial morphology. Gain-of-function missense mutations in *FGFR*s are responsible for craniosynostosis and chondrodysplasia syndromes [28]. Allelic missense mutations of *FGFR2* and *FGFR3* have widely varying phenotypes. The vast majority of mutations that cause craniosynostosis syndromes are associated with the *FGFR2*, which cause quite variable and non-specific phenotypes. As the main role of the *FGFR3* is the regulation of cell proliferation in epiphyseal plate chondrocytes, many *FGFR3* mutations are characterized by short-limbed bone dysplasia of varying severity (hypochondroplasia, achondroplasia, thanatophoric dysplasia); craniosynostosis is rare in the first two of these disorders. Although a reasonable correlation between clinical description and mutation has emerged, identification of these mutations has necessitated some reappraisal of the rather confusing clinical classification of the craniosynostosis disorders. Therefore, abnormalities resulting from mutations in *FGFR2* and *FGFR3* genes are a good example of a disorder that is better classified by mutation rather than phenotype [16,29]. A single missense mutation in *FGFR1* (P252R) can cause Pfeiffer syndrome and phenocopies several Pfeiffer syndrome mutations in *FGFR2* [30]. Moreover, recent studies have identified 22 nonrecurring loss-of- function point mutations in *FGFR1* [31–33]. We focused our studies on the variations of the *FGFR1* gene as the number of mutations already identified in this gene indicates that *FGFR1* might accumulate SNPs that do not result in a pathologic deformation, but may influence normal variation. Furthermore, the *FGFR1* is associated with osteoprogenitor cell differentiation suggesting that mutation in the *FGFR1* gene may affect the function of the differentiated osteobalsts in early embryonic life and hence affect the bone development. Pervious studies have identified a correlation between the SNP rs4647905 of the FGFR1 gene and some head measurements [5,16,17]. Moreover, some reports have highlighted that the SNPs rs13317, rs6996321 and rs881301 may play a crucial role in the association of nonsyndromic clefting and FGFR1 gene [20–22]. Therefore, we suspected that the *FGFR1* SNPs rs881301, rs6996321, rs4647905, and rs13317 might have certain role in the craniofacial morphogenesis. In the present study, we analyzed four *FGFR1* SNPs in Japanese and Korean subjects and observed an association of SNPs rs13317 and rs6996321 with some facial traits, as determined by performing PCA. Interestingly, SNPs rs13317 and rs6996321 were associated with a small face (denoted by PC1) and midfacial retrusion, which in turn resulted in protruded forehead and relatively wide orbit and cheek area (denoted by PC3). These facial features were similar to those of patients with Pfeiffer syndrome, which is caused by a dysfunctional mutation in *FGFR1* [15]. Most facial characteristics of patients with Pfeiffer syndrome result from coronal synostosis with or without sagittal synostosis and thus abnormal growth of the skull bones [15]. This might also be the reason for such craniofacial features in subjects with SNPs rs13317 and rs6996321, which might affect the timing of suture closure and hence craniofacial morphogenesis. Several cephalometric studies reported the various effects of craniosynostosis on the midface region and the mandible in patients with Pfeiffer syndrome. Patients with Pfeiffer syndrome usually have brachycephalic face, high forehead and midfacial retrusion (from the middle of the eye socket to the upper jaw). Moreover, orbitostenosis, characterized by extreme proptosis was evident in in these cases, which is coincident with midfacial retrusion. The mandible of Pfeiffer syndrome patients usually has a shorter body, obtuse gonial angle and changes in the condylar width [15,34–39]. Although our study didn't show any significant correlations between the studied SNPs and the PCs representing the mandibular measurements, significant correlations were observed in some combinations between cranial and mandibular morphological components (S4 Table). The reason behind such results might be that the mandibular deformity usually seen in syndromes accompanied with craniosynostosis is due to cranial base abnormalities and probably not intrinsic to the mandible [38,39].

Our regression analyses by using the stepwise multiple SNP model indicated an association of SNPs rs13317 and rs6996321 with PC1 and PC3. However, the single SNP model showed that only rs13317 was significantly associated with PC1 and that rs6996321 did not show any significant association. Similarly, the single SNP model showed that only rs13317 was significantly associated with the ratio |Or-NA|/Or-Or. These results may be because of the independent effects of at least two polymorphisms in *FGFR1*. Functions of SNPs rs13317 and rs6996321 are unknown. Moreover, it is possible that these SNPs do not have any function other than serving as markers. Because the distance between SNPs rs13317 and rs6996321 is 52.8 kb and because their LD was weak in the analyzed populations, it is likely that effective polymorphisms exist in different LD blocks.

Our results may not be in agreement with those of some previous association studies involving *FGFR1* variants and craniofacial measurements. Coussens and van Daal (2005) observed a significant association between rs4647905 and decreased cephalic index [5]. Moreover, Gòmez-Valdès et al. (2013) reported that rs4647905 was associated with a transversely narrow and elongated anteroposterior head (dolichocephaly) [16], suggesting that this variant played a role in the timing of the closure of skull sutures, particularly the sagittal suture, to establish characteristic craniofacial features. The present study did not measure the cranial index because the range of cephalometric image did not cover the entire cranium. Moreover, rs4647905 was not significantly associated with cranial morphology. However, we observed a significant association of rs4647905 with a mandibular shape pattern. Therefore, further studies should be performed to elucidate the reason underlying the inconsistencies among previous studies. Our study was the first to use lateral and posteroanterior cephalograms to obtain the craniofacial data to examine the association of the *FGFR1* polymorphisms with the normal variation of the craniofacial morphology. However, cephalometric measurements are associated with errors classified as ‘‘errors of projection” and ‘‘errors of identification”. Errors of projection are due to the two- dimensional (2D) head film, which causes a shadow of the three-dimensional (3D) object meaning that complete control is not possible unless the positions of landmarks are known in 3D. The impact of error of projection is mainly on the linear measurements, as the values of the angular measurements remain constant. In addition, those linear measures, which were used, are conventionally defined as lying in the midsagittal plane. In theory, that plane is held at a constant distance from both the film and the emitter by the nature of cephalostat (head holding device) design, and hence all points on it have a similar and standardizable enlargement factor. Precise repositioning of patients in any head holder is, however, very difficult, resulting in a situation in which the true anatomic midsagittal plane coincides with the nominal midsagittal plane of the x-ray-cephalostat system only rarely and by chance as slight head rotation may occur. However, it was found that differences in angular or linear measurements do not become significant unless rotation of the head in the cephalostat exceeds 10 degrees. On the other hand, the recent 3D imaging techniques like the cone beam computed tomography (CBCT) can exactly record and represent the size of the object [40–42]. For that reason, further association studies between the *FGFR1* polymorphisms and the craniofacial morphology is recommended using the modern 3D imaging techniques for more accurate representation of the variations occurring in the craniofacial complex.

In conclusion, we showed that *FGFR1* SNPs played crucial roles in inducing craniofacial morphological variations in Japanese and Korean subjects, primarily because of their effects on the overall size of the head and midfacial development. Subjects with the derived alleles of SNPs rs13317 and rs6996321 had a small face and a facial pattern associated with a retruded midface and relatively wide-set eyes. These facial features were similar to but were milder than those of patients with Pfeiffer syndrome, which is caused by a dysfunctional mutation in *FGFR1*.

## Supporting Information

S1 Table Phenotypes based on cephalometric measurements. (DOCX)

S2 Table Eigenvalues in the cranial PCA. (DOCX)

S3 Table Eigenvalues in the mandibular PCA. (DOCX)

S4 Table Correlation between cranial and mandibular morphological components. (DOCX)
