# Supplementary material for: Contribution of FGFR1 Variants to Craniofacial Variations in East Asians
Source: PLoS One. 2017 Jan 27;12(1):e0170645. doi: 10.1371/journal.pone.0170645 (PMC5271310; doi:10.1371/journal.pone.0170645)
Supplement: S1 Table — (DOCX) [file pone.0170645.s001.docx]

S1 Table. Phenotypes based on cephalometric measurements

|  | All  (n = 443) |  | Japanese men  (n = 43) | | Korean men  (n = 132) | | Male difference | Japanese women  (n = 173) | | Korean women  (n = 95) | | Female difference |
| --- | --- | --- | --- | --- | --- | --- | --- | --- | --- | --- | --- | --- |
|  | Mean | SD | Mean | SD | Mean | SD | P | Mean | SD | Mean | SD | P |
| Cranial measurements |  |  |  |  |  |  |  |  |  |  |  |  |
| Orbital height (mm) | 41.68 | 2.54 | 42.18 | 2.46 | 42.04 | 2.74 | 7.5.E-01 | 41.78 | 2.44 | 40.81 | 2.30 | **1.5.E-03** |
| Latero-orbitale (R)-latero-orbitale (L) (mm) | 99.34 | 4.21 | 99.92 | 4.01 | 102.36 | 3.64 | **8.4.E-04** | 97.25 | 3.59 | 98.70 | 3.56 | **1.8.E-03** |
| Orbitale (R)-orbitale (L) (mm) | 75.79 | 6.61 | 76.33 | 5.09 | 78.96 | 6.30 | **7.5.E-03** | 73.25 | 5.35 | 75.75 | 7.75 | **6.3.E-03** |
| Zygion (R)-zygion (L) (mm) | 138.92 | 8.16 | 138.02 | 6.28 | 147.76 | 5.17 | **6.4.E-13** | 132.32 | 4.80 | 139.15 | 5.14 | **1.1.E-20** |
| Nasal cavity (R)-nasal cavity (L) (mm) | 36.58 | 3.54 | 36.76 | 3.35 | 37.95 | 4.27 | 6.8.E-02 | 36.08 | 3.05 | 35.53 | 2.73 | 1.3.E-01 |
| Nasion-prosthion (mm) | 67.02 | 4.52 | 68.11 | 3.89 | 70.27 | 4.13 | **3.E-03** | 64.56 | 3.68 | 66.48 | 3.72 | **7.E-05** |
| Nasion-condylion (mm) | 89.31 | 5.89 | 88.65 | 4.68 | 95.10 | 4.55 | **2.9.E-11** | 85.31 | 4.03 | 88.78 | 4.18 | **5.6.E-10** |
| Anterior nasal spine-posterior nasal spine (mm) | 52.77 | 4.11 | 52.76 | 5.25 | 55.51 | 3.36 | **2.1.E-03** | 50.68 | 3.63 | 52.78 | 2.93 | **6.3.E-07** |
| Point A-condylion (mm) | 92.03 | 5.84 | 93.54 | 6.31 | 96.55 | 5.08 | **6.1.E-03** | 88.68 | 4.67 | 91.13 | 3.93 | **9.3.E-06** |
| Upper incisor length (mm) | 25.95 | 2.61 | 25.99 | 2.60 | 26.87 | 2.25 | 5.1.E-02 | 25.35 | 2.60 | 25.76 | 2.77 | 2.3.E-01 |
| Glabellare-NA plane (mm) | 3.85 | 2.13 | 5.02 | 2.18 | 5.27 | 2.10 | 5.1.E-01 | 2.81 | 1.61 | 3.22 | 1.58 | **4.8.E-02** |
| Supra orbitale-NA plane (mm) | -4.58 | 2.06 | -4.44 | 2.18 | -4.56 | 2.11 | 7.5.E-01 | -4.40 | 1.86 | -5.02 | 2.26 | **2.4.E-02** |
| Rhinion-NA plane (mm) | 12.76 | 2.58 | 13.69 | 2.80 | 13.62 | 2.48 | 8.8.E-01 | 12.09 | 2.35 | 12.35 | 2.56 | 4.1.E-01 |
| Orbitale-NA plane (mm) | -11.32 | 2.62 | -12.74 | 2.47 | -11.96 | 2.85 | 9.0.E-02 | -10.92 | 2.22 | -10.50 | 2.58 | 1.9.E-01 |
| Key ridge-NA plane (mm) | -24.19 | 3.27 | -24.18 | 4.33 | -25.28 | 3.16 | 1.3.E-01 | -23.40 | 2.99 | -24.12 | 2.98 | 6.3.E-02 |
| Mandibular measurements |  |  |  |  |  |  |  |  |  |  |  |  |
| Condylion (R)-condylion (L) (mm) | 116.89 | 8.05 | 114.17 | 6.38 | 124.36 | 7.07 | **3.8.E-13** | 111.40 | 5.05 | 117.73 | 5.73 | **5.3.E-16** |
| Koronion (R)-koronion (L) (mm) | 110.05 | 6.93 | 109.94 | 7.18 | 114.51 | 6.72 | **5.3.E-04** | 107.54 | 5.97 | 108.49 | 5.75 | 2.1.E-01 |
| Gonion (R)-gonion (L) (mm) | 110.79 | 8.06 | 111.13 | 6.23 | 118.26 | 6.90 | **1.9.E-08** | 105.30 | 5.45 | 110.28 | 5.82 | **1.4.E-10** |
| Lower incisor length (mm) | 22.60 | 2.31 | 23.87 | 2.38 | 22.78 | 2.27 | **1.0.E-02** | 22.99 | 2.19 | 21.06 | 1.78 | **2.3.E-13** |
| Infradentale-gonion (mm) | 82.91 | 6.71 | 83.25 | 5.87 | 88.61 | 5.43 | **1.4.E-06** | 77.99 | 5.05 | 83.76 | 4.16 | **1.0.E-19** |
| Infradentale-gnathion (mm) | 33.58 | 3.38 | 35.11 | 3.68 | 35.36 | 2.97 | 6.8.E-01 | 32.45 | 3.15 | 32.45 | 2.82 | 1.0.E+00 |
| Gnathion-gonion (mm) | 127.88 | 8.56 | 136.10 | 9.02 | 133.51 | 6.00 | 8.4.E-02 | 123.40 | 7.60 | 124.39 | 5.15 | 2.1.E-01 |
| Gnathion-condylion (mm) | 82.58 | 6.35 | 84.32 | 6.31 | 87.22 | 5.16 | **8.2.E-03** | 78.40 | 5.33 | 82.95 | 4.29 | **1.1.E-12** |
| Gonion-condylion (mm) | 66.12 | 7.07 | 70.36 | 5.12 | 71.79 | 5.10 | 1.2.E-01 | 61.80 | 6.26 | 64.14 | 4.85 | **8.4.E-04** |
| PCs |  |  |  |  |  |  |  |  |  |  |  |  |
| Cranium PC1 | 0.00 | 2.13 | 0.48 | 1.49 | 2.29 | 1.48 | **4.5.E-12** | -1.62 | 1.37 | -0.37 | 1.24 | **2.6.E-07** |
| Cranium PC2 | 0.00 | 1.28 | -0.07 | 1.28 | 0.03 | 1.25 | 7.1.E-01 | 0.01 | 1.05 | -0.03 | 1.25 | 8.3.E-01 |
| Cranium PC3 | 0.00 | 1.24 | 0.06 | 1.25 | 0.33 | 1.30 | 1.8.E-01 | -0.18 | 0.87 | -0.15 | 1.25 | 9.4.E-01 |
| Cranium PC4 | 0.00 | 1.03 | -0.37 | 0.83 | 0.05 | 1.11 | **3.5.E-02** | -0.15 | 0.76 | 0.38 | 1.03 | **6.5.E-05** |
| Cranium PC5 | 0.00 | 1.02 | 0.40 | 0.80 | -0.14 | 0.93 | **1.8.E-03** | 0.20 | 0.90 | -0.35 | 0.95 | **3.2.E-05** |
| Cranium PC6 | 0.00 | 0.97 | 0.32 | 0.82 | 0.17 | 0.93 | 6.1.E-01 | -0.16 | 0.79 | -0.07 | 1.02 | 6.8.E-01 |
| Mandible PC1 | 0.00 | 2.12 | 0.77 | 1.53 | 2.14 | 1.52 | **4.2.E-09** | -1.65 | 1.37 | -0.36 | 1.14 | **1.9.E-08** |
| Mandible PC2 | 0.00 | 1.07 | -0.94 | 1.03 | 0.17 | 1.00 | **9.8.E-07** | -0.31 | 0.79 | 0.76 | 0.82 | **4.2.E-18** |
| Mandible PC2 | 0.00 | 1.00 | -0.50 | 0.90 | 0.19 | 0.94 | **2.6.E-04** | 0.09 | 0.91 | -0.20 | 0.78 | **9.8.E-03** |

Bold, P < 0.05.Bol LD coefficients hisms are present in different LD blocks.
