# Supplementary material for: Contribution of FGFR1 Variants to Craniofacial Variations in East Asians
Source: PLoS One. 2017 Jan 27;12(1):e0170645. doi: 10.1371/journal.pone.0170645 (PMC5271310; doi:10.1371/journal.pone.0170645)
Supplement: S2 Table — (DOCX) [file pone.0170645.s002.docx]

S2 Table. Eigenvalues in the cranial PCA

|  | Eigenvalue | Contribution | Cumulative contribution |
| --- | --- | --- | --- |
| Cranial PC1 | 4.549 | 30.32% | 30.32% |
| Cranial PC2 | 1.644 | 10.96% | 41.28% |
| Cranial PC3 | 1.532 | 10.21% | 51.49% |
| Cranial PC4 | 1.068 | 7.12% | 58.62% |
| Cranial PC5 | 1.036 | 6.91% | 65.52% |
| Cranial PC6 | 0.940 | 6.27% | 71.79% |
| Cranial PC7 | 0.790 | 5.27% | 77.06% |
| Cranial PC8 | 0.742 | 4.95% | 82.00% |
| Cranial PC9 | 0.593 | 3.95% | 85.96% |
| Cranial PC10 | 0.536 | 3.57% | 89.53% |
| Cranial PC11 | 0.463 | 3.08% | 92.61% |
| Cranial PC12 | 0.402 | 2.68% | 95.30% |
| Cranial PC13 | 0.325 | 2.16% | 97.46% |
| Cranial PC14 | 0.238 | 1.59% | 99.05% |
| Cranial PC15 | 0.143 | 0.95% | 100.00% |
