# Supplementary material for: Contribution of FGFR1 Variants to Craniofacial Variations in East Asians
Source: PLoS One. 2017 Jan 27;12(1):e0170645. doi: 10.1371/journal.pone.0170645 (PMC5271310; doi:10.1371/journal.pone.0170645)
Supplement: S3 Table — (DOCX) [file pone.0170645.s003.docx]

S3 Table. Eigenvalues in the mandibular PCA

|  | Eigenvalue | Contribution | Cumulative contribution |
| --- | --- | --- | --- |
| Mandibular PC1 | 4.476 | 49.73% | 49.73% |
| Mandibular PC2 | 1.150 | 12.78% | 62.51% |
| Mandibular PC3 | 0.993 | 11.03% | 73.54% |
| Mandibular PC4 | 0.669 | 7.44% | 80.98% |
| Mandibular PC5 | 0.641 | 7.12% | 88.10% |
| Mandibular PC6 | 0.458 | 5.09% | 93.19% |
| Mandibular PC7 | 0.316 | 3.51% | 96.70% |
| Mandibular PC8 | 0.250 | 2.77% | 99.47% |
| Mandibular PC9 | 0.047 | 0.53% | 100.00% |
