# Supplementary material for: Contribution of FGFR1 Variants to Craniofacial Variations in East Asians
Source: PLoS One. 2017 Jan 27;12(1):e0170645. doi: 10.1371/journal.pone.0170645 (PMC5271310; doi:10.1371/journal.pone.0170645)
Supplement: S4 Table — (DOCX) [file pone.0170645.s004.docx]

S4 Table. Correlation between cranial and mandibular morphological components

|  |  | Mandibular PC1 | Mandibular PC2 | Mandibular PC3 |
| --- | --- | --- | --- | --- |
| Cranial PC1 | R | **0.844** | 0.016 | **0.098** |
|  | P | **3.8.E-116** | 7.4.E-01 | **4.4.E-02** |
| Cranial PC2 | R | 0.018 | -0.013 | **0.182** |
|  | P | 7.2.E-01 | 8.0.E-01 | **1.7.E-04** |
| Cranial PC3 | R | 0.090 | **0.164** | **0.173** |
|  | P | 6.4.E-02 | **6.8.E-04** | **3.5.E-04** |
| Cranial PC4 | R | 0.056 | **0.222** | -0.032 |
|  | P | 2.5.E-01 | **3.9.E-06** | 5.1.E-01 |
| Cranial PC5 | R | -0.016 | -0.049 | **-0.106** |
|  | P | 7.4.E-01 | 3.1.E-01 | **2.9.E-02** |
| Cranial PC6 | R | **0.132** | -0.004 | **-0.148** |
|  | P | **6.6.E-03** | 9.4.E-01 | **2.2.E-03** |

R, correlation coefficient; P, P value; bold, P < 0.05.
